# Burnout Syndrome in Paediatric Nurses: A Multi-Centre Study

**DOI:** 10.3390/ijerph18031324

**Published:** 2021-02-01

**Authors:** Emilia I. De la Fuente-Solana, Laura Pradas-Hernández, Carmen Tamara González-Fernández, Almudena Velando-Soriano, María Begoña Martos-Cabrera, José L. Gómez-Urquiza, Guillermo Arturo Cañadas-De la Fuente

**Affiliations:** 1 Brain, Mind and Behavior Research Center (CIMCYC), Campus Universitario de Cartuja s/n, University of Granada, 18071 Granada, Spain; edfuente@ugr.es; 2 San Cecilio Clinical University Hospital, Andalusian Health Service, Avenida de la Investigación s/n, 18016 Granada, Spain; begomartos90@gmail.com; 3 Virgen de las Nieves University Hospital, Andalusian Health Service, Avenida de las Fuerzas Armadas, nº6, 18014 Granada, Spain; carmentamara09@gmail.com (C.T.G.-F.); srtavelando@gmail.com (A.V.-S.); 4 Faculty of Health Sciences, University of Granada, Avenida de la Ilustración 60, 18016 Granada, Spain; jlgurquiza@ugr.es (J.L.G.-U.); gacf@ugr.es (G.A.C.-D.l.F.)

**Keywords:** burnout, nursing, paediatrics, personality factors, prevention

## Abstract

Background: Burnout syndrome is an increasingly prevalent problem, characterised by emotional exhaustion (EE), depersonalization (D), and low personal accomplishment (PA), feelings that appear with prolonged exposure to stress-inducing situations. The syndrome alters physical well-being and endangers the quality of services provided. Among nurses working in the paediatric area, the association between burnout and the corresponding risk profile has received little research attention, despite the highly stressful nature of this work. Materials and Methods: The study population was composed of 95 nurses working in four hospitals in the province of Granada. Data were collected using the Maslach Burnout Inventory, the NEO Personality Inventory, and the Educational-Clinical Questionnaire: Anxiety and Depression. Results: According to the results obtained, 22.0% of the nurses working in the paediatric area present high levels of EE, 18.5% present high levels of D, and 39.6% had feelings of low PA. These burnout levels do not depend on sociodemographic or labour variables, but the three domains of the syndrome are related to the psychological factors analysed. Conclusions: Among the nurses who participated in this study, 38.6% presented high levels of burnout, especially regarding feelings of low personal accomplishment. Personality factors play an important role in the development of this syndrome. This study shows the impact of burnout in paediatric nurses as well as the risk factors, providing information for the development of strategies to prevent it.

## 1. Introduction

Burnout syndrome is an increasingly prevalent problem. One sector of the working population where burnout is particularly acute is that of healthcare professionals, among which nurses are especially vulnerable [1].

Burnout arises from constant exposure to pressure in the workplace, which provokes a negative impact both on the persons affected and on their environment [2]. Many definitions of burnout have been proposed, but one of the most widely accepted is that of Maslach and Jackson (1981), who described burnout as a psychological syndrome that arises in response to chronic stressors, and is characterized by three dimensions—emotional exhaustion (EE), depersonalization (D), and low personal accomplishment (PA). Moreover, this initial view emphasized that burnout affected individuals whose work involved interaction with others [1,3,4,5].

On this conceptual basis, the above-mentioned authors created the Maslach Burnout Inventory (MBI), which measured the same three domains and was intended to quantify the degree of burnout suffered—low, medium, or high [6]. Although other validated scales with the same objective have been suggested, and multiple adaptations have been made for different population groups [7,8,9], the MBI is still one of the most commonly used [10].

Burnout is considered an occupational disease. It can produce multiple psychological problems, such as anxiety and depression, leading those affected to abandon the profession [2], thus having negative consequences both for nurses and for the work environment. Nurses are especially exposed to the danger of burnout, as they must often work in adverse conditions with few resources [11]. Moreover, if nurses suffer from burnout, the care provided will also suffer, in terms of productivity and healthcare quality, to the detriment of patients [12].

Among the nursing community, those employed in the paediatric area, caring for patients aged 0–15 years, are at great risk of developing significant levels of burnout, because of the working conditions implicit in their profession, which impose both physical and emotional demands [13,14,15,16]. Studies have shown that in the Andalusian Health Service (SAS), the prevalence of burnout is extreme in various hospital services, including the medical department [17], oncology [18], critical care [19], and primary care [20]. Nevertheless, to our knowledge, no research information has been published concerning burnout among paediatric nurses.

In view of these considerations, the present study has the following aims: to determine the prevalence and levels of burnout experienced by nurses in the paediatric area of SAS hospitals; to identify the degree of burnout among paediatric nurses, according to the model proposed by Golembiewski; and to describe the relationship between burnout and the relevant sociodemographic, occupational, and personality factors.

## 2. Materials and Methods

### 2.1. Sample

In this cross-sectional multicenter study, the sample population consisted of 95 nurses working in the paediatric units of four high-level public hospitals in the province of Granada (Spain), forming part of the Andalusian Health Service. Study data were collected using non-random or convenience sampling by quotas. In this procedure, questionnaires were distributed in sealed envelopes to the target population. Of the 130 distributed, 98 were returned, of which 95 were complete and suitable for analysis.

### 2.2. Study Design and Data Collection

Data for this study were collected with the collaboration of the nursing management and the service supervisors of each hospital. The hospitals were given the necessary information and the requisite permissions were obtained. The nurses received detailed documentation about the study, and gave signed informed consent to participate. Participation was voluntary, individual, and anonymous. The estimated time required to complete the questionnaire was 45 min. Collection of the study data began in October 2019 and concluded in February 2020.

The questionnaire consisted of several parts. In the first, the following sociodemographic and occupational data were obtained: on the one hand, age (years), sex (male/female), marital status (single, married, separated, divorced, or widowed) and children (n); and on the other, work shift (rotating/fixed), on-call obligation (yes/no), and nursing experience (months).

The Revised NEO Personality Inventory [21] (NEO-FFI), Spanish-language version [22], was applied. This instrument measures the five major personality traits—neuroticism, extraversion, conscientiousness, agreeableness, and openness. Our study used a shorter version of the inventory, with 60 items, 12 for each domain, scored on a five-point Likert scale. As a result of copyright, the full questionnaire is not presented in this paper. The following internal consistency coefficients were obtained: neuroticism (α = 0.729), extraversion (α = 0.737), conscientiousness (α = 0.84), agreeableness (α = 0.629), and openness (α = 0.715). Cronbach’s alpha (α) is used for assessing the reliability of a questionnaire (internal consistency). It must be calculated in the study sample in order to verify that the reliability of the original questionnaire is maintained.

In addition, the Educational-Clinical Questionnaire: Anxiety and Depression (CECAD) [23] was used to measure symptoms of anxiety and depression, in accordance with the Diagnostic and Statistical Manual of Mental Disorders (DSM-V). This instrument consists of 45 items scored on a five-point Likert scale, with 19 items for anxiety and 26 for depression. The internal consistency coefficients obtained were α = 0.934 for anxiety and α = 0.954 for depression.

The three domains of burnout were measured using the Maslach Burnout Inventory-Human Service Survey (MBI-HSS) [6], in the Spanish-language version [9], which consists of 22 items, scored on a seven-point Likert scale. This questionnaire contains nine items on EE, five on D, and eight on PA. The cut-off points (established by the authors and included in the MBI manual) at which each domain was considered to represent a high level of burnout were >24 for EE, >9 for D, and <33 for PA. A low level of EE is <15 points and <4 points for D. A medium level of EE is between 15–24 points and between 4–9 for D. A medium PA is between 33–39 points and high PA is > than 39. The internal consistency coefficients obtained were α = 0.861 for EE, α = 0.628 for D, and α = 0.758 for PA [20].

Finally, the overall level of burnout was obtained according to the process described by Golembiewski and Munzenrider [24], and the score for each burnout domain was classified as low or high, via eight phases. Subjects with low or high burnout in the three dimensions obtain a low or high burnout phase classification, respectively. Medium phases are the result of combining different levels in the dimensions, starting with an increase in D and later in low PA and EE. With this data, Golembiewski et al. obtained significant differences for concluding that phases 1 and 2 are low burnout; phases 3, 4, and 5 are moderate burnout; and 6, 7, and 8 are high burnout [24].

### 2.3. Statistical Analysis

The following descriptive statistics were obtained for the quantitative variables: mean, standard deviation, and maximum and minimum values. For the qualitative variables, percentages and frequencies were obtained. The quantitative variables (EE, D, and PA) in relation to the qualitative variables considered, and were compared using Student’s t-test. Pearson’s correlation coefficient was used to determine the linear relationships between the quantitative variables. Finally, a multiple linear regression analysis was performed for each of the MBI domains. All of the analyses were performed using SPSS 25.0 statistical software (IBM, Armonk, NY, USA).

### 2.4. Ethical Considerations

In all cases, participation in this study was voluntary, anonymous, and conducted in accordance with the ethical guidelines of the Declaration of Helsinki. In addition, the study was approved by the Granada Provincial Research Ethics Committee (TSS-SD-2019).

## 3. Results

### 3.1. Demographic Profile

The study sample was composed of 95 nurses working in paediatric departments. By sex, 78.7% were female and 21.3% were male. On average, the participants were 43.89 years old, and had been working in the paediatric area for 156.92 months and overall as nurses for 254.97 months.

Table 1 and Table 2 show the descriptive variables of the sample and the mean scores obtained for the three domains of burnout (EE, D, and PA), the five dimensions of personality (neuroticism, extraversion, conscientiousness, agreeableness, and openness), and the parameters of anxiety and depression.

### 3.2. Levels and Prevalence of Burnout

To determine the levels of burnout, the score obtained in each domain was categorised as low, medium, or high, in accordance with the cut-off points proposed in the Spanish-language version of the MBI [20].

The application of this method produced the following results: in EE, 46.2% of the participating nurses presented a low level of burnout, 31.8% a moderate level, and 22.0% a high level. In D, 50.0% presented a low level, 31.5% a moderate level, and 18.5% a high level. Finally, in PA, 39.6% presented a low level, 31.8% a moderate level, and 28.6% a high level. Table 3 shows the information about burnout levels.

### 3.3. Phases of Burnout Syndrome

The Golembiewski model [24] was used to classify the participants by phases, according to the level of burnout presented. Phases I, II, and III represent low levels of burnout; phases IV and V, medium levels; and phases VI, VII and VIII, high levels.

In summary, 38.6% of the nurses who took part in this study presented high levels of burnout (Table 4).

### 3.4. Relationship between Burnout and Sociodemographic and Occupational Factors

The mean values for each MBI domain were compared by referencing the sociodemographic and occupational variables considered (sex, marital status, number of children, work shift, and on-call obligation). In these respects, no significant differences were observed. Similarly, no significant relationships were obtained between occupational factors (age and experience in the paediatric area and in the nursing profession) and burnout.

### 3.5. Relationship between Burnout and Psychological Factors: Explanatory Models

A linear correlation was observed between the MBI domains, those of the NEO-FFI, and the CECAD anxiety and depression scores.

The EE and D domains presented statistically significant correlations with anxiety and depression, as well as with the personality factors, except that of openness.

PA was significantly correlated with depression, but not with anxiety. All other domains, including openness, presented a statistically significant correlation (Table 5).

A multiple linear regression model was obtained for each of the burnout domains. The model explains 46.7% of the variability of EE (r2 = 0.467), for which both depression (B = 0.264; *p* = 0.000) and neuroticism (B = 0.547; *p* = 0.001) were statistically significant predictors. The goodness of fit value for D was r^2^ = 0.324. The variables that were statistically significant predictors of this domain were depression (B = 0.067; *p* = 0.021), conscientiousness (b = −0.188; *p* = 0.023), and agreeableness (B = −0.288; *p* = 0.002). Finally, for PA, 31% of the variability was explained by the model (r^2^ = 0.310). In this case, conscientiousness (B = 0.503; *p* = 0.000) and extraversion (B = 0.337; *p* = 0.009) were statistically significant predictors (Table 6).

## 4. Discussion

The results obtained show that 22.0% of the paediatric nurses surveyed presented high levels of EE, 18.5% high levels of D, and 39.6% low levels of PA. These levels of EE and D are greater than those shown in one study with nurses from critical care units [19], lower than another study with primary care nurses [20], and also show a higher prevalence of low PA and a lower prevalence of EE than those shown in a meta-analysis with paediatric oncology nurses [16].

These levels show that a significant amount of paediatric nurses are affected by burnout, corroborating previous research findings [25,26,27], and may be related to the generally low levels of job satisfaction perceived by these professionals. When this occurs, it not only has negative repercussions on the healthcare workers themselves, but may also lead them to feel less empathy with their patients and/or fail to perform their work appropriately, resulting in a decrease in the quality of care provided [28,29]. High levels of burnout can be influenced by various characteristics, including marital status, age, professional experience, and the burden of care [25].

Studies have observed a relationship between burnout and certain sociodemographic and occupational characteristics. Some have suggested that age is inversely related to the syndrome, i.e., that younger nurses are more likely to experience this condition [30,31]. Other papers, however, have drawn the opposite conclusion, finding that older workers are more likely to perceive low levels of PA [32]. By sex, D has been reported to be more acute among male than female nurses [33], and regarding marital status, it has been reported that single nurses have lower levels of PA, which may reflect inadequate forms of coping with adversity or a perceived lack of social support [34]. One study found the length of professional experience to be inversely related to burnout; in other words, relatively inexperienced nurses are more likely to suffer from this syndrome [30]. However, we found no relationship between the burnout domains and the sociodemographic variables considered (age, marital status, and children) or those concerning the work environment (seniority and shift or on-call obligation), which is in line with some previous findings [35]. We also corroborate results showing that these variables do not appear to significantly influence levels of burnout. In other words, neither the nurses’ age, gender, nor length of experience determine their exposure to burnout; this is particularly evident in high-risk areas, such as paediatric oncology [16,36,37,38].

Regarding the prevalence of burnout in paediatric nurses, the values we obtained for PA, EE, and D were higher than those reported previously [39]. EE is especially high among nurses working in the oncology area, while high D and low PA are more prevalent in paediatric departments [40]. This contrast is mainly due to the types of patients treated in the different departments; patients may remain in oncology treatment for extended periods and the staff become more emotionally involved, a consequence of which can be high levels of EE.

When establishing risk profiles for nurses working in the paediatric area, psychological variables must be considered. For example, EE and personality factors are often interrelated; in our study population, neuroticism was found to be associated with greater EE, as observed previously by Ang et al. [41]. Moreover, it is inversely associated with extraversion, conscientiousness, agreeableness, and openness [42]. It has also been observed that elevated levels of EE are related to greater anxiety and depression, because of these nurses’ daily exposure to traumatic stressors in their work environment [43].

Similarly, D is often associated with personality factors. Thus, our findings reflect a positive association with neuroticism and a negative one with extraversion, conscientiousness, agreeableness, and openness, suggesting that professionals with this type of personality take a closer, more empathetic approach in their work [42,44]. This domain is also positively related to depression and anxiety, which implies that nurses may feel insecure regarding patient care and the attendant risks. For this reason, some professionals try not to become closely involved, which in turn favors D [45].

Finally, an inverse relationship was observed between PA and neuroticism, and a positive one with the other personality factors [46]. Depression and anxiety also presented an inverse relationship with PA. The high levels of PA recorded among the nurses in this study and the fact that 38.6% presented high levels of burnout may be related to factors such as emotional burden, arising from close contact with children and their families; with the disease process; and, in some cases, with the death of the patient [12]. The joint impact of these factors, together with the scant personal and professional recognition often received, can generate attitudes of indifference among paediatric nurses, and can even lead them to consider abandoning the profession [47,48]. Previous studies in this field have concluded that low PA is related to a lack of professional autonomy [49], together with other factors, such as low salary [50].

In our study, the regression models obtained show that the personality variables, with the exception of openness, and the level of depression experienced, are significant predictors of burnout. These results are in line with those obtained in studies of other medical services [47], that 22.0% of the paediatric nurses surveyed present high levels of EE, 18.5% high levels of D, and 39.6% low PA.

### 4.1. Applications in Clinical Practice

The results obtained in the present study highlight the importance of implementing strategies and interventions at an early stage in order to protect workers from burnout [51,52], through greater social support or initiatives focused on mindfulness [47,53].

### 4.2. Limitations

This study is subject to certain limitations. In the first place, although it did include staff from four different hospitals, the study sample was not very large, the sampling was intentional, and nursed feeling some level of burnout might have been more prone to participate. Secondly, although various personality variables were considered, others, such as resilience, were omitted and could usefully have been analysed. Furthermore, in future work in this field, attention should be paid to the question of stress in healthcare. It would be convenient to study what other variables could be part of a burnout risk profile. More specifically, it is of interest which variables can reduce or protect nurses from burnout. Finally, although the Golembiewski model used is very useful as a classification tool, further research is needed to develop methods based on measuring physiological parameters, in order to analyse them more precisely and to establish a better basis for diagnosing burnout syndrome.

## 5. Conclusions

Many nurses working in the paediatric area experience high levels of burnout. In this respect, the question of scant personal accomplishment is the domain most often cited by these workers.

Among the psychological variables considered, neuroticism and depression were found to be related to a greater vulnerability to burnout, while agreeableness, extraversion, conscientiousness, and openness protect against it. In the case of anxiety, it affects EE and D, but not PA levels. The sociodemographic and occupational factors considered were not significantly associated with the propensity to burnout. These results raise the importance of creating strategies and programs for promoting burnout protecting factors and improving the problem of burnout in this area.

## Figures and Tables

**Table 1:** Descriptive data and qualitative variables.

Variable		% (n)
Sex	Male	23.2 (22)
	Female	76.8 (73)
Marital status	Single	30.5 (29)
	Married	62 (59)
	Separated	5.3 (5)
	Divorced	1.1 (1)
	Widowed	1.1 (1)
Children	None	27.7 (26)
	One	18.1 (17)
	Two	40.4 (38)
	Three or more	13.8 (13)
Work shift	Fixed-morning	17.9 (17)
	Fixed-afternoon/evening	2.1 (2)
	Fixed-night	2.1 (2)
	Rotating	77.9 (74)
On-call	Yes	33 (30)
	No	67 (61)

**Table 2:** Descriptive data and quantitative variables.

Variable		Mean (SD)	Minimum–Maximum
Age, years (n = 94)		43.89 (10.500)	22–61
Experience in paediatrics, months (n = 95)		156.92 (135.004)	1–432
Experience in profession, months (n = 95)		254.97 (129.976)	2–480
**NEO-FFI**	Neuroticism (n = 94)	27.98 (6.849)	1–48
	Extraversion (n = 95)	42.51 (6.240)	29–58
	Openness (n = 94)	38.07 (6.539)	25–55
	Agreeableness (n = 94)	46.53 (5.045)	34–60
	Conscientiousness (n = 94)	47.36 (6.007)	33–59
**CECAD**	Anxiety (n = 93)	35.69 (11.860)	19–89
	Depression (n = 92)	50.02 (16.686)	26–116
**MBI**	EE (n = 91)	16.37 (10.651)	0–47
	D (n = 92)	5.15 (4.806)	0–21
	PA (n = 91)	35.93 (7.376)	14–48

**Table 3:** Categorisation of levels of burnout by domain.

Burnout Level	EE % (n)	D % (n)	PA % (n)
Low	46.2 (42)	50.0 (46)	39.6 (36)
Moderate	31.8 (29)	31.5 (29)	31.8 (29)
High	22.0 (20)	18.5 (17)	28.6 (26)

**Table 4:** Prevalence of burnout by phases according to the Golembiewski model.

Phase	I	II	III	IV	V	VI	VII	VIII
**EE**	L	L	L	L	H	H	H	H
**D**	L	H	L	H	L	H	L	H
**PA**	L	L	H	H	L	L	H	H
***N***	9	7	29	5	4	15	11	8
**(%)**	10.2	8.0	33.0	5.7	4.5	17.0	12.5	9.1

EE—emotional exhaustion; D—depersonalisation; PA—personal accomplishment; H—high; L—low.

**Table 5:** Coefficient of correlation between psychological variables and burnout.

		EE	D	PA
NEO-FFI	Neuroticism	0.610 **	0.372 **	−0.285 **
	Extraversion	−0.407 **	−0.351 **	0.422 **
	Conscientiousness	−0.318 **	−0.441 **	0.497 **
	Agreeableness	−0.276 **	−0.439 **	0.269 *
	Openness	−0.149	−0.132	0.280 **
CECAD	Depression	0.637 **	0.409 **	−0.236 *
	Anxiety	0.572 **	0.292 **	−0.153

EE—emotional exhaustion; D—depersonalisation; PA—personal accomplishment; * = *p* < 0.001; ** = *p* < 0.005.

**Table 6:** Multiple linear regression between the burnout domains, personality factors, anxiety, and depression.

	B	Std. Error	Beta	*t*	*p*
**EE**					
**Depression**	0.264	0.068	0.404	3.864	0.000
**Neuroticism**	0.547	0.163	0.350	3.347	0.001
**D**					
**Depression**	0.067	0.029	0.231	2.358	0.021
**Conscientiousness**	−0.188	0.081	−0.236	−2.312	0.023
**Agreeableness**	−0.288	0.091	−0.305	−3.154	0.002
**RP**					
**Conscientiousness**	0.503	0.129	0.391	3.903	0.000
**Extraversion**	0.337	0.126	0.268	2.675	0.009

EE—emotional exhaustion; D—depersonalisation; PA—personal accomplishment; B—estimated parameter; *t*—Student’s t-test value.

## Data Availability

The data presented in this study are available on request from the corresponding author. The data are not publicly available due to ethical.

## References

[1] Schaufeli W.B., Leiter M.P., Maslach C. (2009). Burnout: 35 Years of Research and Practice. Career Dev. Int..

[2] Iserson K.V. (2018). Burnout Syndrome: Global Medicine Volunteering as a Possible Treatment Strategy. J. Emerg. Med..

[3] Maslach C. (1982). Burnout: The Cost of Caring.

[4] Maslach C., Schaufeli W.B., Leiter M.P. (2001). Job Burnout. Annu. Rev. Psychol..

[5] Maslach C., Jackson S.E., Leiter M. (2016). Maslach Burnout Inventory Manual.

[6] Maslach C., Jackson S.E. (1981). The Measurement of Experienced Burnout. J. Organ. Behav..

[7] De la Fuente E.I., García J., Cañadas G.A., San Luis C., Cañadas G.R., Aguayo R., de la Fuente L., Vargas C. (2015). Psychometric Properties and Scales of the Granada Burnout Questionnaire Applied to Nurses. Int. J. Clin. Health Psychol..

[8] De La Fuente E.I., Lozano L.M., García-Cueto E., Luis C.S., Vargas C., Cañadas G.R., Cañadas-De la Fuente G.A., Hambleton R.K. (2013). Development and Validation of the Granada Burnout Questionnaire in Spanish Police. Int. J. Clin. Health Psychol..

[9] Seisdedos N. (1997). Manual Del Inventario Burnout de Maslach. Síndrome Del “Quemado” Por Estrés Laboral Asistencial.

[10] Halbesleben J.R.B., Demerouti E. (2005). The construct validity of an alternative measure of burnout: Investigating the English translation of the Oldenburg Burnout Inventory. Work Stress.

[11] Membrive-Jiménez M.J., Pradas-Hernández L., Suleiman-Martos N., Vargas-Román K., Cañadas-De la Fuente G.A., Gomez-Urquiza J.L., De la Fuente-Solana E.I. (2020). Burnout in Nursing Managers: A Systematic Review and Meta-Analysis of Related Factors, Levels and Prevalence. Int. J. Environ. Res. Public Health.

[12] Arrogante O., Aparicio-Zaldivar E. (2017). Burnout and Health among Critical Care Professionals: The Mediational Role of Resilience. Intensive Crit. Care Nurs..

[13] Omayda-Urbina-Laza C. (2012). La Enfermería Pediátrica En Los Cuidados Para La Salud Infantil. Rev. Cubana Enferm..

[14] Quintanilla M. (2004). Prevalencia Del Sindrome de Burnout En Las Enfermeras de La Unidad de Paciente Críticos Del Hospital Del Trabajador Santiago de Chile y Una Propuesta de Intervención. Rev. Chil. Med. Intensiva.

[15] Pradas-Hernández L., Ariza T., Gómez-Urquiza J.L., Albendín-García L., De la Fuente E.I., Cañadas-De la Fuente G.A. (2018). Prevalence of burnout in paediatric nurses: A systematic review and meta-analysis. PLoS ONE.

[16] De la Fuente-Solana E.I., Pradas-Hernández L., Ramiro-Salmerón A., Suleiman-Martos N., Gómez-Urquiza J.L., Albendín-García L., Cañadas-De la Fuente G.A. (2020). Burnout Syndrome in Paediatric Oncology Nurses: A Systematic Review and Meta-Analysis. Healthcare.

[17] Ramirez-Baena L., Ortega-Campos E., Gomez-Urquiza J.L., Cañadas-De la Fuente G.R., De la Fuente-Solana E.I., Cañadas-De la Fuente G.A. (2019). A Multicentre Study of Burnout Prevalence and Related Psychological Variables in Medical Area Hospital Nurses. J. Clin. Med..

[18] De la Fuente-Solana E.I., Cañadas G.R., Ramirez-Baena L., Gómez-Urquiza J.L., Ariza T., Cañadas-De la Fuente G.A. (2019). An Explanatory Model of Potential Changes in Burnout Diagnosis According to Personality Factors in Oncology Nurses. Int. J. Environ. Res. Public Health.

[19] Cañadas-De la Fuente G.A., Albendín-García L., Cañadas G.R., San Luis-Costas C., Ortega-Campos E., De la Fuente-Solana E.I. (2018). Nurse Burnout in Critical Care Units and Emergency Departments: Intensity and Associated Factors. Emergencias.

[20] Ortega-Campos E., Cañadas-De la Fuente G.A., Albendín-García L., Gómez-Urquiza J.L., Monsalve-Reyes C., de la Fuente-Solana E.I. (2019). A Multicentre Study of Psychological Variables and the Prevalence of Burnout among Primary Health Care Nurses. Int. J. Environ. Res. Public Health.

[21] Costa P.T., McCrae R.R. (1989). The NeoPI/Neo-FFI Manual Supplement.

[22] Costa P., McCrae R.R. (2002). Inventario de Personalidad NEO Revisado. Inventario NEO Reducido de Cinco Factores (NEO-FFI).

[23] Lozano L., García-Cueto E., Lozano L. (2007). Cuestionario Educativo Clínico de Ansiedad y Depresión.

[24] Golembiewski R.T., Munzenrider R., Praeger Publishers (1988). Phases of Burnout: Developments in Concepts and Applications.

[25] Akman O., Ozturk C., Bektas M., Ayar D., Armstrong M.A. (2016). Job Satisfaction and Burnout among Paediatric Nurses. J. Nurs. Manag..

[26] Kalliath T., Morris R. (2002). Job Satisfaction among Nurses: A Predictor of Burnout Levels. J. Nurs. Adm..

[27] Rosales R.A., Rosales G.L., Labrague L.J. (2013). Nurses’ Job Satisfaction and Burnout: Is There a Connection?. Int. J. Adv. Nurs. Stud..

[28] Braithwaite M. (2008). Nurse Burnout and Stress in the NICU. Adv. Neonatal Care.

[29] Adwan J.Z. (2014). Pediatric Nurses’ Grief Experience, Burnout and Job Satisfaction. J. Pediatr. Nurs..

[30] Hunter B., Fenwick J., Sidebotham D.M., Henley D.J. (2019). Midwives in the United Kingdom: Levels of Burnout, Depression, Anxiety and Stress and Associated Predictors. Midwifery.

[31] Cagan O., Gunay O. (2015). The Job Satisfaction and Burnout Levels of Primary Care Health Workers in the Province of Malatya in Turkey. Pakistan J. Med. Sci..

[32] Buckley L., Berta W., Cleverley K., Medeiros C., Widger K. (2020). What Is Known about Paediatric Nurse Burnout: A Scoping Review. Human Resources for Health.

[33] Hatch D.J., Freude G., Martus P., Rose U., Müller G., Potter G.G. (2018). Age, Burnout and Physical and Psychological Work Ability among Nurses. Occup. Med..

[34] Mefoh P.C., Ude E.N., Chukwuorji J.B.C. (2019). Age and Burnout Syndrome in Nursing Professionals: Moderating Role of Emotion-Focused Coping. Psychol. Health Med..

[35] Xian M., Zhai H., Xiong Y., Han Y. (2020). The Role of Work Resources between Job Demands and Burnout in Male Nurses. J. Clin. Nurs..

[36] Vega P.V., Rodriguez R.G., Galdamez N.S., Molina C.F., Orellana J.S., Villanueva A.S., Melo J.B. (2017). Supporting in Grief and Burnout of the Nursing Team from Pediatric Units in Chilean Hospitals. Rev. Esc. Enferm..

[37] López Franco M., Rodríguez Núñez A., Fernández Sanmartín M., Marcos Alonso S., Martinón Torres F., Martinón Sánchez J.M. (2005). Síndrome de Desgaste Profesional En El Personal Asistencial Pediátrico. An. Pediatr..

[38] Hu H.X., Liu L.T., Zhao F.J., Yao Y.Y., Gao Y.X., Wang G.R. (2015). Factors Related to Job Burnout among Community Nurses in Changchun, China. J. Nurs. Res..

[39] De la Fuente-Solana E.I., Suleiman-Martos N., Velando-Soriano A., Cañadas-De la Fuente G.R., Herrera-Cabrerizo B., Albendín-García L. (2020). Predictors of Burnout of Health Professionals in the Departments of Maternity and Gynaecology, and Its Association with Personality Factors: A Multicentre Study. J. Clin. Nurs..

[40] Cañadas-De la Fuente G.A., Gómez-Urquiza J.L., Ortega-Campos E.M., Cañadas G.R., Albendín-García L., De la Fuente-Solana E.I. (2018). Prevalence of Burnout Syndrome in Oncology Nursing: A Meta-Analytic Study. Psycho-Oncology.

[41] Ang S.Y., Dhaliwal S.S., Ayre T.C., Uthaman T., Fong K.Y., Tien C.E., Zhou H., Della P. (2016). Demographics and Personality Factors Associated with Burnout among Nurses in a Singapore Tertiary Hospital. Biomed. Res. Int..

[42] Toh S.G., Ang E., Devi M.K. (2012). Systematic Review on the Relationship between the Nursing Shortage and Job Satisfaction, Stress and Burnout Levels among Nurses in Oncology/Haematology Settings. Int. J. Evid. Based Healthc..

[43] Favrod C., du Chêne L.J., Soelch C.M., Garthus-Niegel S., Tolsa J.F., Legault F., Briet V., Horsch A. (2018). Mental Health Symptoms and Work-Related Stressors in Hospital Midwives and NICU Nurses: A Mixed Methods Study. Front. Psychiatry.

[44] Cañadas-De la Fuente G.A., Vargas C., San Luis C., García I., Cañadas G.R., De la Fuente E.I. (2015). Risk Factors and Prevalence of Burnout Syndrome in the Nursing Profession. Int. J. Nurs. Stud..

[45] De la Fuente-Solana E.I., Gómez-Urquiza J.L., Cañadas G.R., Albendín-García L., Ortega-Campos E., Cañadas-De la Fuente G.A. (2017). Burnout and Its Relationship with Personality Factors in Oncology Nurses. Eur. J. Oncol. Nurs. Off. J. Eur. Oncol. Nurs. Soc..

[46] Geuens N., Braspenning M., Van Bogaert P., Franck E. (2015). Individual Vulnerability to Burnout in Nurses: The Role of Type D Personality within Different Nursing Specialty Areas. Burn. Res..

[47] Hastings-Tolsma M., Foster S.W., Brucker M.C., Nodine P., Burpo R., Camune B., Griggs J., Callahan T.J. (2018). Nature and Scope of Certified Nurse-Midwifery Practice: A Workforce Study. J. Clin. Nurs..

[48] Shi R., Zhang S., Xu H., Liu X., Miao D. (2015). Regulatory Focus and Burnout in Nurses: The Mediating Effect of Perception of Transformational Leadership. Int. J. Nurs. Pract..

[49] Galindo R.H., de Oliveira Feliciano K.V., dos Santos Lima R.A., de Souza A.I. (2012). Síndrome de Burnout Entre Enfermeiros de Um Hospital Geral Da Cidade Do Recife. Rev. Esc. Enferm..

[50] Ruotsalainen J.H., Verbeek J.H., Mariné A., Serra C., Marine A., Serra C. (2014). Preventing Occupational Stress in Healthcare Workers. Cochrane Database Syst. Rev..

[51] Suleiman-Martos N., Gomez-Urquiza J.L., Aguayo-Estremera R., Cañadas-De la Fuente G.A., De La Fuente-Solana E.I., Albendín-García L. (2020). The Effect of Mindfulness Training on Burnout Syndrome in Nursing: A Systematic Review and Meta-Analysis. J. Adv. Nurs..

[52] Bloxsome D., Ireson D., Doleman G., Bayes S. (2019). Factors Associated with Midwives’ Job Satisfaction and Intention to Stay in the Profession: An Integrative Review. J. Clin. Nurs..

[53] Velando-Soriano A., Ortega-Campos E., Gómez-Urquiza J.L., Ramírez-Baena L., De La Fuente E.I., Cañadas-De La Fuente G.A. (2020). Impact of Social Support in Preventing Burnout Syndrome in Nurses: A Systematic Review. Jpn. J. Nurs. Sci..

